# Supplementary material for: Uptake of COVID-19 vaccine among high-risk urban populations in Southern Thailand using the COM-B model
Source: PLoS One. 2024 Mar 14;19(3):e0300509. doi: 10.1371/journal.pone.0300509 (PMC10939195; doi:10.1371/journal.pone.0300509)
Supplement: Attachment — Submitted filename: Rebuttal Letter.docx [file pone.0300509.s002.docx]

Dear Editors,

We would like to thank the editor for their thoughtful evaluation of our initial manuscript entitled ***Uptake of COVID-19 vaccine among high-risk urban populations in Southern Thailand using the COM-B Model.*** Please find our revised manuscript, which we believe is substantially strengthened now that we have incorporated editor’s’ recommendations.

Editors’ comments:

Please, find a point-by-point response to the editors’ comments below.

1. After internal review of your manuscript, we had several concerns with analyses, conclusions, and quality of the scientific reporting. Please note that as per our publication criteria, PLOS ONE requires that all experiments, statistics, and other analyses be performed to a high technical standard, described in sufficient detail and adhere to appropriate reporting guidelines and community standards. Conclusions must be presented in the appropriate fashion and be supported by the data. After careful consideration, we feel that current manuscript does not meet these requirements.

**Response:**

Thank you so much. We appreciate your important suggestions based on the initial version of our manuscript submitted to PLOS ONE. In this version, we have provided more details to statistics and analyses. The current manuscript is reported according to appropriate reporting guidelines. Conclusions are supported by data. We believe that the current version of the manuscript is substantially strengthened to meet the criteria’s for PLOS review.

2. For example, you have not provided us with sufficient information regarding the steps taken to fully validate the questionnaire used for the data collection in the study. The methodology and results from the reliability and pilot testing on the questionnaire have not been reported robustly.

**Response:**

Thank you so much. We have now provided sufficient information regarding the steps taken to validate the questionnaire and as well as on the reliability and pilot testing of the questionnaire.. It now reads “The questionnaire, which consisted of sections on socio-demographic information, COVID-19 vaccine-related information, and the COM-B model, was initially drafted in English and later translated into the local language (Thai) for the ease of administration. Before the commencement of the study, we pretested the questionnaire in another Facebook group with 65 participants to check and revise (if required) its verbal consistency. Additionally, we checked the structure reliability of the questionnaire in the pretested sample, and the Cronbach’s Alpha value (Thai version) for capability, opportunity, motivation, and total was 0.86, 0.77, 0.82, and 0.83, respectively. We have also provided the details to the sections of the questionnaire under the sections measures.

3. Sufficient details were not provided on the sampling approach and eligibility criteria utilized during the participant recruitment stage to enable replication of your study.

**Response:**

Thank you so much. We presented more details to sample size calculation, sampling and eligibility criteria. The sample calculation and sampling now reads “The estimated sample size was 325, obtained by a sample size calculation from the Facebook group HATY AIZ records, which documented the number of active adult members (800 members; July –August 2021) with a 95% confidence level, a 5% margin of error, a design effect of 1.5, and a 10% nonresponse rate. We increased the calculated sample size from a minimum of 325 participants to 379 participants in the prevision of missing data. The final analysis consists of 358 participants with their complete data sets.”

We used convenience sampling (voluntary participation) to recruit our participants in this study.

We have added details to the eligibility criteria for recruitment of our study participants. It now reads “We recruited a total of 358 participants who were currently active members of the Facebook group HATY AIZ to participate in an anonymous survey using the online Google survey forms via Facebook Messenger. Our participants mainly lived in cities, were young, spoke Thai, and were willing to participate in this study.”

4. Due to this limitation we are unable to evaluate whether the conclusions of the study presented are generalizable to wider population.

**Response:**

Thank you so much. Given the aforementioned reasons, the findings of this study will aid future public health interventions in Thailand and beyond.

5. Additionally, our second publication criterion notes, “if a submitted study replicates or is very similar to previous work, authors must provide a sound scientific rationale for the submitted work and clearly reference and discuss the existing literature. Submission that replicate or are derivative of existing work will likely be rejected if the authors do not provide adequate justification.” In the case of your submission, we do not feel that you have sufficiently discussed the related literature within its topic and highlight how in relation to those studies, the current manuscript further contributes to scientific knowledge.

**Response:**

Thank you so much for the suggestion. We have now provided more details to the existing knowledge and also addressed the importance of our study in the introduction section and as well as have discussed them in the discussed sections.

In the introduction section, we have added details as “ There is growing recognition that theory-based behavioral models effectively promote COVID-19 prevention behaviors [19-21]. The capability, opportunity, motivation, and behavior (COM-B) model refers to one of the effective models in preventing COVID-19 disease and acted as a practical framework for designing and promoting preventive behaviors [20]. This behavioral model includes the constructs of capability, opportunity, and motivation. The results of several studies indicate that the application of this model is successful in COVID-19 prevention [22-23]. There is little knowledge in the study area of the COM-B model, influencing the uptake of COVID-19 vaccines.  To understand how individuals’ behavioral intentions toward COVID-19 vaccine influence their decision to uptake the COVID-19 vaccine, we carried out a web-based cross-sectional study based on the COM-B model. The findings of this study will be of immense importance in formulating strategies for the enhancement of COVID-19 vaccination in Thailand and beyond.”

We have added details to the discussion section and it now reads “ It is well known that theory-based behavioral models are crucial in adopting a behavior [19-22,32-33]. We observed that 69.6%, 41.9%, and 12% of our study participants, respectively, had a high level of capability, opportunity, and motivation toward the uptake of the COVID-19 vaccine based on the COM-B model. In addition, we found that participants with high levels of opportunity and motivation were significantly more likely to receive their first dose of the COVID-19 vaccine than participants with low levels of opportunity and motivation. Several studies reported that factors that influence behavioral intentions toward the COVID-19 vaccine are multi-dimensional [28-29]. A study in Thailand found that the constructs in the COM-B model could successfully generate themes of behaviors that affect COVID-19 prevention intentions [22]. Other studies have revealed that the adapted COM-B model provides refined details for each construct in the vaccine context [21,23]. Poor capability, motivation, and opportunity often restrict people from the care or services they require. The need for effective interventions designed for the individual and contextual constructs of the COM-B model in the COVID-19 vaccine context has never been greater.

6. Furthermore, PLOS ONE has been specifically designed for the publication of the results for of primary scientific research that must be addressed in the standard English and the language must be clear, correct, and unambiguous. In this case, we do not feel that your submission meets this criterion: your manuscript requires extensive editing for English grammar and usage, as well as over all scientific readability.

**Response:**

Thank you so much. We have re-written the whole manuscript to meet standards sets for scientific publications. We will further utilize editing services if our manuscript is accepted for publication.

We would like to thank you once again for the initial evaluation of our manuscript and we believe that our revised manuscript is substantially strengthened now that we have incorporated editors’ comments. We hope that our responses will be acceptable to the editors and our manuscript will be marked eligible for further review.
