# Supplementary material for: Uptake of COVID-19 vaccine among high-risk urban populations in Southern Thailand using the COM-B model
Source: PLoS One. 2024 Mar 14;19(3):e0300509. doi: 10.1371/journal.pone.0300509 (PMC10939195; doi:10.1371/journal.pone.0300509)
Supplement: Attachment — Submitted filename: SRIRATH-SENT1112023.docx [file pone.0300509.s003.docx]

**Uptake of COVID-19 vaccine among high-risk urban populations in Southern Thailand using the COM-B Model**

**Srirath Gohwong’s comment**

1. The research complies with ethical standards for experimentation and research integrity, as it was granted approval by Walailak University's Ethics Committee (reference number WUEC-21-214-01, dated August 16, 2021). Informed consent was diligently secured from all participants through an official online consent form, as provided by the committee. This form transparently communicated the study's goals, objectives, and the participants' entitlement to withdraw at any point. Furthermore, the study steadfastly adhered to the ethical principles outlined in the Declaration of Helsinki, which stands as one of its outstanding strengths.

2. The title of this study, "Enhancing COVID-19 Vaccine Uptake in High-Risk Urban Populations in Southern Thailand through the Application of the COM-B Model," boldly showcases its unique and innovative approach. The assessment for potential plagiarism conducted by Chulalongkorn University resulted in a favorable outcome, revealing a similarity index of 0.00% (Source: [http://plag.grad.chula.ac.th], [http://www.chula.ac.th/en/]). This academic research demonstrates exceptional originality and quality, and the plagiarism assessment findings reinforce my confidence in its academic integrity.

3. Is the work clearly and accurately presented and does it cite the current literature?

The work does cite a range of current literature relevant to the research topic. The references included in the provided text encompass a variety of sources, including academic journals, international organizations like the World Health Organization (WHO), and primary research studies. The references cover a wide range of topics related to the COVID-19 pandemic, vaccination, and factors influencing vaccine acceptance.

4. Is the study design appropriate and does the work have academic merit? Are Experiments, statistics, and other analyses performed to a high technical standard and are described in sufficient detail?

The study design is well-suited to the research objectives, involving a web-based cross-sectional study conducted in Hat Yai district, an urban area in southern Thailand significantly affected by the COVID-19 pandemic. The study's focus on COVID-19 vaccine uptake among high-risk urban populations is justified. The measures, including socio-demographic characteristics and the adapted Capability, Opportunity, Motivation, and Behavior (COM-B) model, are academically sound. The data collection process is well-documented, with pretesting and reliability checks. The statistical analysis using SPSS, descriptive statistics, and multivariable logistic regression aligns with the study's objectives. The use of a significance level (P < .05) is appropriately stated. However, providing more detail on specific statistical tests, control variables, and effect sizes would enhance the analysis, especially multivariable logistic regression. Logistic multiple regression is a statistical approach employed when analyzing a binary outcome variable, such as yes/no or 1/0, in relation to multiple independent variables (predictors or covariates). The author should reveal the specific conditions and assumptions of Logistic multiple regression: the outcome must be binary, observations should be independent, a linear relationship between predictors and log-odds is assumed, multicollinearity should be avoided, a sufficient sample size is needed, outliers need attention, perfect separation can pose challenges, model fit must be evaluated, odds ratios should be appropriately interpreted, and causal relationships should be established through experimental designs or advanced statistical methods. Adhering to these considerations is vital for ensuring the robustness and reliability of results. Collaboration with a statistician or data analyst is recommended for addressing complex modeling issues and assessing potential assumption violations. The limitations of the study are well-acknowledged, including the smaller sample size, potential under-representation of certain demographic groups, and the fact that the study focused on a particular period of the pandemic. The study's recognition of these limitations demonstrates a thorough understanding of the research's scope and potential constraints.

5. If applicable, is the statistical analysis and its interpretation appropriate?

The statistical analysis and interpretation of the data are generally appropriate. The study presents a comprehensive analysis of the socio-demographic characteristics of the participants, their capability, opportunity, motivation, and the factors associated with COVID-19 vaccine uptake. The use of descriptive statistics, such as percentages and means, is suitable for presenting the data. The logistic regression analysis effectively identifies factors associated with vaccine uptake. However, there is room for minor improvements. While the results are well presented, it would be beneficial to include the confidence intervals for the percentages and means to indicate the precision of the estimates. Moreover, the interpretation of the logistic regression results could be more detailed, explaining the adjusted odds ratios and their implications more explicitly. In conclusion, the study's analysis and interpretation are sound, but enhancing clarity and precision in presenting results and their implications would further strengthen the paper.

6. Are the conclusions drawn adequately supported by the results? [4. Conclusions are presented in an appropriate fashion and are supported by the data.]

The study's conclusions find robust support in the data and analyses. It reports a COVID-19 vaccine uptake rate of 59.5% among participants, backed by the data's clear portrayal of vaccination status and construct scores for capability, opportunity, and motivation. Furthermore, the study uncovers various demographic factors linked to vaccine acceptance, such as marital status, education, and employment, as indicated by the odds ratios derived from multivariable logistic regression analysis. Notably, the study underscores the effectiveness of the COM-B model's opportunity and motivation constructs in driving vaccine uptake, leveraging comprehensive data from Tables 2, 3, and 4. It wisely calls for future research encompassing larger and more diverse populations to validate these findings and offers guidance on evidence-based interventions for enhancing COVID-19 vaccine uptake based on the identified factors and COM-B constructs.
