# Supplementary material for: Uptake of COVID-19 vaccine among high-risk urban populations in Southern Thailand using the COM-B model
Source: PLoS One. 2024 Mar 14;19(3):e0300509. doi: 10.1371/journal.pone.0300509 (PMC10939195; doi:10.1371/journal.pone.0300509)
Supplement: Attachment — Submitted filename: Response to Reviewers.docx [file pone.0300509.s004.docx]

Dear Editor,

We would like to thank the editor and the reviewers for their thoughtful evaluation of our manuscript entitled **“Uptake of COVID-19 vaccine among high-risk urban populations in Southern Thailand using the COM-B Model”.** Please find our revised manuscript along with tracked changes file (yellow highlighted), which we believe is substantially strengthened now that we have incorporated reviewers’ recommendations.

**Journal requirements:**

We made amendments according to the journal requirements.

**Additional Editor Comments:**

Overall, this manuscript was written quite well and received very positive feedback from reviewers. If this manuscript is submitted for consideration at the end of 2021 or early 2022, it will need to be accepted and published quickly as it is a study to face the urgent situation at that time. However, now that the COVID-19 situation has passed and interest in vaccination has decreased a lot because it is not something that people still don't know even a little bit about it that might be helpful. Looking at the details of this manuscript, I found that these are issues that many studies have already addressed. This is especially true when considering the population of this study that is not unique or may provide new, deeper or more interesting answers.

**Response:** Thank you so much for your positive comments. We would like to add the findings of this study could be of significant importance in adopting a new behavior through application of the COM-B model in any given disaster. We have provided more details for the practical implications of our key findings (yellow highlighted in the manuscript).

To make this manuscript interesting and useful for publication at this time, the authors need to improve this manuscript in at least two parts.

1. Point out how important and interesting the southern region of Thailand is within a context of a developing country. However, there should also be strong empirical data to support it.

**Response:** Thank you so much. We agree with your comment and added details in the introductions section and methodology, and pointed out this point through out the discussion of our revised manuscript (Please see lines 95-102, lines 106-112, please see discussion).

2. If it is not possible to change the independent variables used in the study, the authors should not only point out what variables are present but also explain the interesting points from the discovery of the relationship between those variables. This is not only to answer questions about COVID-19, but also to deal with future outbreaks in this type of area. This means that the authors need to be more explicit about the issues being studied and how they are more important or interesting than in many previous studies. Moreover, it is a discussion and expansion to show how the results of this research can be further developed to obtain answers that are more useful than existing research.

**Response:** Thank you so much. We are indeed very thankful for this feedback. We have revised most sections of our manuscript and addressed this important issue of how our findings can be important in future events like COVID-19 disease. Moreover, we have expanded our discussion of how our findings can be useful (Please see lines 338-340, lines 343-345, lines 364-369, lines 372-375, lines 380-383, lines 394-397, and lines 411-415). We have also highlighted key findings for policy consideration. Thank you once again and we really hope that our manuscript would be acceptable to you.

**Response to Reviewers’ comments**

**Response to Reviewer 1 comments**

I have no concerns about the issue of republishing. This research is a new research study in the context of the southern region of Thailand, and as research ethics standards certified by the host university. In addition, this research also writes useful research results that will lead to future public health guidelines in Thailand and countries with similar contexts.

**Response:** Thank you so much for recommending the publication of our manuscript.

**Response to Reviewer 2 comments**

1. The study is ethically sound, receiving approval from Walailak University's Ethics Committee and obtaining informed consent from participants. The research demonstrates exceptional originality, as confirmed by a favorable plagiarism assessment. It appropriately references current literature, covering a wide range of topics related to COVID-19 and vaccination. The study design, a web-based cross-sectional approach in a high-risk urban area, aligns with its objectives and uses academically sound measures.

**Response:** Thank you so much for constructive and positive feedback.

2. Statistical analysis and interpretation are generally appropriate, though presenting confidence intervals for percentages and means and providing more detailed interpretation of logistic regression results could enhance clarity.

**Response:** Thank you so much for this feedback. We have added 95% CI for COVID-19 vaccines percentages, added means for COM-B constructs, and added p-value to the logistic regression results (Please see the results).

3. Overall, the conclusions are well supported by data and analyses, highlighting a 59.5% vaccine uptake rate and identifying demographic factors and the influence of the COM-B model on vaccine acceptance. The study suggests further research and provides valuable insights for evidence-based interventions.

**Response:** Thank you so much.

**Responses to reviewer 2 detailed feedback.**

1. The research complies with ethical standards for experimentation and research integrity, as it was granted approval by Walailak University's Ethics Committee (reference number WUEC-21-214-01, dated August 16, 2021). Informed consent was diligently secured from all participants through an official online consent form, as provided by the committee. This form transparently communicated the study's goals, objectives, and the participants' entitlement to withdraw at any point. Furthermore, the study steadfastly adhered to the ethical principles outlined in the Declaration of Helsinki, which stands as one of its outstanding strengths.

**Response:** Thank you so much.

2. The title of this study, "Enhancing COVID-19 Vaccine Uptake in High-Risk Urban Populations in Southern Thailand through the Application of the COM-B Model," boldly showcases its unique and innovative approach. The assessment for potential plagiarism conducted by Chulalongkorn University resulted in a favorable outcome, revealing a similarity index of 0.00% (Source: [http://plag.grad.chula.ac.th], [http://www.chula.ac.th/en/]). This academic research demonstrates exceptional originality and quality, and the plagiarism assessment findings reinforce my confidence in its academic integrity.

**Response:** Thank you so much for positive feedback.

3. Is the work clearly and accurately presented and does it cite the current literature?

The work does cite a range of current literature relevant to the research topic. The references included in the provided text encompass a variety of sources, including academic journals, international organizations like the World Health Organization (WHO), and primary research studies. The references cover a wide range of topics related to the COVID-19 pandemic, vaccination, and factors influencing vaccine acceptance.

**Response:** Thank you so much.

4. Is the study design appropriate and does the work have academic merit? Are Experiments, statistics, and other analyses performed to a high technical standard and are described in sufficient detail?
   a. The study design is well-suited to the research objectives, involving a web-based cross-sectional study conducted in Hat Yai district, an urban area in southern Thailand significantly affected by the COVID-19 pandemic. The study's focus on COVID-19 vaccine uptake among high-risk urban populations is justified. The measures, including socio-demographic characteristics and the adapted Capability, Opportunity, Motivation, and Behavior (COM-B) model, are academically sound. The data collection process is well documented, with pretesting and reliability checks. The statistical analysis using SPSS, descriptive statistics, and multivariable logistic regression aligns with the study's objectives. The use of a significance level (P < .05) is appropriately stated.

**Response:** Thank you so much.

b. However, providing more detail on specific statistical tests, control variables, and effect sizes would enhance the analysis, especially multivariable logistic regression. Logistic multiple regression is a statistical approach employed when analyzing a binary outcome variable, such as yes/no or 1/0, in relation to multiple independent variables (predictors or covariates). The author should reveal the specific conditions and assumptions of Logistic multiple regression: the outcome must be binary, observations should be independent, a linear relationship between predictors and log-odds is assumed, multicollinearity should be avoided, a sufficient sample size is needed, outliers need attention, perfect separation can pose challenges, model fit must be evaluated, odds ratios should be appropriately interpreted, and causal relationships should be established through experimental designs or advanced statistical methods. Adhering to these considerations is vital for ensuring the robustness and reliability of results. Collaboration with a statistician or data analyst is recommended for addressing complex modeling issues and assessing potential assumption violations.

**Response:** Thank you so much. We agree with your comments and have added the suggested details. In our methods, we have declared that all assumptions for multivariable logistic regression analysis were met (Please see line 177-178).

c. The limitations of the study are well-acknowledged, including the smaller sample size, potential under-representation of certain demographic groups, and the fact that the study focused on a particular period of the pandemic. The study's recognition of these limitations demonstrates a thorough understanding of the research's scope and potential constraints.

**Response:** Thank you so much for the positive feedback.

5. If applicable, are the statistical analysis and its interpretation appropriate?
   a. The statistical analysis and interpretation of the data are generally appropriate. The study presents a comprehensive analysis of the socio-demographic characteristics of the participants, their capability, opportunity, motivation, and the factors associated with COVID-19 vaccine uptake. The use of descriptive statistics, such as percentages and means, is suitable for presenting the data. The logistic regression analysis effectively identifies factors associated with the vaccine uptake.

**Response:** Thank you so much.

b. However, there is room for minor improvements. While the results are well presented, it would be beneficial to include the confidence intervals for the percentages and means to indicate the precision of the estimates. Moreover, the interpretation of the logistic regression results could be more detailed, explaining the adjusted odds ratios and their implications more explicitly. In conclusion, the study's analysis and interpretation are sound, but enhancing clarity and precision in presenting results and their implications would further strengthen the paper.

**Response:** Thank you so much for the constructive feedback. We have added the suggested points to the results (please see the results section). Moreover, we have detailed the implications of our findings in the discussion section (Please see lines 338-340, lines 343-345, lines 364-369, lines 372-375, lines 380-383, lines 394-397, and lines 411-415).

6. Are the conclusions drawn adequately supported by the results? [Conclusions are presented in an appropriate fashion and are supported by the data.]

The study's conclusions find robust support in the data and analyses. It reports a COVID-19 vaccine uptake rate of 59.5% among participants, backed by the data's clear portrayal of vaccination status and construct scores for capability, opportunity, and motivation. Furthermore, the study uncovers various demographic factors linked to vaccine acceptance, such as marital status, education, and employment, as indicated by the odds ratios derived from multivariable logistic regression analysis. Notably, the study underscores the effectiveness of the COM-B model's opportunity and motivation constructs in driving vaccine uptake, leveraging comprehensive data from Tables 2, 3, and 4. It wisely calls for future research encompassing larger and more diverse populations to validate these findings and offers guidance on evidence-based interventions for enhancing COVID-19 vaccine uptake based on the identified factors and COM-B constructs.

**Response:** Thank you so much for clarifying these important findings and providing positive feedback to our manuscript.

At last, we would like to thank respected editor and respected reviewers for the overall positive feedback on our manuscript and hope that it will be acceptable to them.

*Sincerely,*
