# Supplementary material for: Uptake of COVID-19 vaccine among high-risk urban populations in Southern Thailand using the COM-B model
Source: PLoS One. 2024 Mar 14;19(3):e0300509. doi: 10.1371/journal.pone.0300509 (PMC10939195; doi:10.1371/journal.pone.0300509)
Supplement: Attachment — Submitted filename: GOIINC18022024.docx [file pone.0300509.s005.docx]

Title: Uptake of COVID-19 vaccine among high-risk urban populations in Southern Thailand using the COM-B Model

Reviewer: **Srirath G., Ph.D.**

1. The study, titled "Uptake of COVID-19 vaccine among high-risk urban populations in Southern Thailand using the COM-B Model," stands out for its absolute absence of plagiarism, verified by Chulalongkorn University with a similarity index of 0.00%. The research's remarkable uniqueness enhances trust in its academic integrity.

2. The study, titled "Uptake of COVID-19 vaccine among high-risk urban populations in Southern Thailand using the COM-B Model," stands out for its absolute absence of plagiarism, verified by Chulalongkorn University with a similarity index of 0.00%. The research's remarkable uniqueness enhances trust in its academic integrity. Obtaining approval from Walailak University's Ethics Committee and securing formal online consent from participants ensures ethical research conduct, emphasizing a commitment to safeguarding participant rights and well-being. This approach, reinforced by an approved consent form containing detailed study information and alignment with the Declaration of Helsinki's ethical principles, enhances transparency, credibility, and trust within the academic community.

3. The grant [WU-COE-66-16] from the Excellent Center for Dengue and Community Public Health at Walailak University plays a pivotal role in encouraging the study`s execution by providing essential resources and creating a conducive environment for rigorous research, data collection, and analysis. This financial support significantly contributes to the overall success and impact of the study within the field of public health.

4. Is the work clearly and accurately presented and does it cite the current literature?

The study, titled "Uptake of COVID-19 vaccine among high-risk urban populations in Southern Thailand using the COM-B Model," provided a clear and accurate presentation, utilizing a web-based cross-sectional approach in the Hat Yai district, Southern Thailand. It strengthened its findings by drawing on a diverse array of contemporary literature, encompassing socio-economic implications, global vaccine acceptance rates, and factors influencing vaccine acceptance. Through an extensive citation of current literature, the research established a robust contextual foundation, weaving insights from reviews on socio-economic impacts, paradigms of vaccine development, and studies on vaccine acceptance rates into its analytical framework. The comprehensive and up-to-date engagement with relevant sources enhanced the study's credibility, providing a well-supported backdrop for its research findings.

5. Is the study design appropriate and does the work have academic merit? Are Experiments, statistics, and other analyses performed to a high technical standard and are described in sufficient detail?

The study on COVID-19 vaccine uptake in Southern Thailand employed a web-based cross-sectional design in the Hat Yai district, recruiting 358 participants from the HATY AIZ Facebook group during September and October 2021. The well-calculated sample size, based on active adult members, incorporated a 95% confidence level, a 5% margin of error, a design effect of 1.5, and a 10% nonresponse rate. Utilizing a structured questionnaire and rigorous statistical methods, including descriptive statistics and multivariable logistic regression analysis in SPSS version 21.00, the study demonstrated a high technical standard in its design, data collection, and analyses, contributing to its academic merit. However, the author was required to illustrate the fulfillment of all prerequisites for employing multivariable logistic regression analysis to confirm the satisfaction of three foundational assumptions. These included ensuring independence among observations, preventing perfect multicollinearity among independent variables, and establishing a linear relationship between continuous predictors and a transformed version of the outcome (linearity).

6. If applicable, is the statistical analysis and its interpretation appropriate?

The study, titled "Uptake of COVID-19 vaccine among high-risk urban populations in Southern Thailand using the COM-B Model," conducted a web-based cross-sectional analysis in the Hat Yai district, involving 358 participants from the HATY AIZ Facebook group during September and October 2021. The results highlighted demographic characteristics, revealing that 59.5% of participants had received at least one dose of the COVID-19 vaccine. The participants, mainly aged 18-60, demonstrated varying levels of capability, opportunity, and motivation regarding COVID-19 vaccine acceptance. The results indicated that being married, having a graduate degree, regular employment, high opportunity, and high motivation were associated with a higher likelihood of COVID-19 vaccine uptake, as determined by multivariable logistic regression analysis. The study identified factors influencing vaccine acceptance and highlighted the importance of theory-based behavioral models in understanding and addressing vaccination intentions, including marital status, education, employment, high opportunity, and high motivation. However, the study acknowledged limitations, including a relatively small sample size, potential under-representation of certain demographic groups in the online survey, and the need for further validation of the COM-B model's psychometric properties in the context of COVID-19 vaccination.

7. Are the conclusions drawn adequately supported by the results?

The study's conclusions were well-supported by the presented results, with detailed descriptive statistics aiding in understanding the study sample. The reported vaccination rate of 59.5% aligned with previous studies, and factors influencing vaccine uptake, identified through multivariable logistic regression, were consistent with existing literature. The application of the COM-B model and recommendations for further research highlighted the study's contribution to understanding and improving COVID-19 vaccine uptake, providing valuable insights for public health interventions.
