# Supplementary material for: Uptake of COVID-19 vaccine among high-risk urban populations in Southern Thailand using the COM-B model
Source: PLoS One. 2024 Mar 14;19(3):e0300509. doi: 10.1371/journal.pone.0300509 (PMC10939195; doi:10.1371/journal.pone.0300509)
Supplement: Attachment — Submitted filename: Response to Reviewers.docx [file pone.0300509.s006.docx]

Dear Editor,

We would like to thank the editor and the reviewers for their positive evaluation of our manuscript entitled **“Uptake of COVID-19 vaccine among high-risk urban populations in Southern Thailand using the COM-B Model”.** Please find our revised manuscript along with tracked changes file (yellow highlighted), which we believe is substantially strengthened now that we have incorporated editorial recommendations.

**Journal requirements:**

We made amendments according to the journal requirements.

**Additional Editor Comments:**

Thank you for the revision and taking the efforts to address all comments. To further improve the manuscript's readability, we would like to invite you to address the below.

**Response:** We also would like to thank the respected editor and reviewers for their approval of our responses and revision. Each point was very helpful for making our paper better and suitable for publication in your prestigious journal. As such, we have tried to amend the manuscript in light of all your and reviewers’ comments and the journal guideline. Please find our responses to the editorial requests.

1. Abstract" Add in the total number of sample size after the word the word Participant= Results: In this study, females constituted 54.7% of the total participants (n=XX)...

**Response:** Thank you so much for you’re your constructive feedback. We have added the sample size (please see page: 2, line: 29).

2. Given these findings, vaccination polices and strategies specific to populations with single/separated marital status for future pandemics are crucial, particularly considering the **likelihood of lower social support** in such cases. This is not clear in term of what do you meant by likelihood of lower social support and how is it related to the single/separated marital status?

**Response:** Thank you. We agree with you that this section was not clear and revised as suggested (please see lines 295 and 296).

3. Please format the tables according to PLOS ONE requirement.

**Response:** Thank you. We formatted our tables according to PLOS ONE requirement.

4. Please attached a letter of proofread of the entire revised manuscript.

**Response:** Thank you so much for this suggestion. All authors re-edited the manuscript to remove the grammatical errors and improve the manuscript. Besides, we have sent the manuscript to English language expert in US (our colleague) and she has proofread it. Please let us know if any error remains.

5. Please help to provide at short 1 line on "HATY AIZ"- what is HATY AIZ?

**Response:** Thank you so much. We agree with your suggestion and have added a line on HATY AIZ group (please see line 113).

6. In the procedure section, please state the right to withdraw as well as token of appreciation- if applicable.

**Response:** Thank you. In our research, participants had the right to withdraw at any stage and we have added these details into the procedure section (please see lines 163 and 185).

7. Please change all p-value in upper case e.g., in here "statistical significance at **P < 0.05**" (line 178, abstract) to lower case and italic "*p <0.05*"

**Response:** Thank you so much. We made changes as suggested (please see line 26, line 175, and Table 5 heading).

8. Seeking your elaboration on Capability, Opportunity, Motivation, and Behavior. In line 145, you mentioned that "In each scale, a score of >10 was used to signify a high level of capability' but in line 204 and 205, it was also stated that "The mean score of the capability construct was 9.53 (± 1.55 SD) with a range of 3-12 points, and 205 nearly two-thirds (249; 69.6%) of them showed a high level of capability". 9.53 certainly is not >10. Hence, I am not sure how did you arrive at the conclusion that "205 nearly two-thirds (249; 69.6%) of them showed a high level of capability". Same concern on "Opportunity to receive COVID-19 vaccine". Can you please clarify this for better readability?

**Response:** Thank you so much for your meticulous observation. We checked our descriptive statistics again and found that there was some error in typing and the mean scores have been corrected now. We have also checked our other analyses and the findings were correctly typed in other cases, except this section which was highlighted by the editor. Additionally the cut-off score should have been written ≥ 10 (typo mistake). We have made these corrections too. Please see the changes on lines 143, 208, 217, and 231.

9. Please include this article in your in text citation - line 78 to support the claim on "trust in healthcare professionals" - <https://bmcpublichealth.biomedcentral.com/articles/10.1186/s12889-022-12632-z>  (citation requests are optional)

**Response:** Thank you so much for this suggestion. We have added this relevant citation into our manuscript.

**Response to Reviewers’ comments**

**Response to Reviewer 1 comments**

**Comment:** It is a particularly interesting study that studies the use of COVID-19 vaccines. In groups High-risk urban populations in southern Thailand. The model is integrated opportunity, motivation, and behavior (COM-B Model). The findings are detailed comprehensive and beneficial to Thailand's public health in the future. It can be used to plan policy measures on intervention measures in the event of an outbreak similar to COVID-19.

**Response:** Thank you so much for your constructive feedback and recommendation on the publication of our manuscript.

**Response to Reviewer 2 comments**

**Comment:** The study, titled "Uptake of COVID-19 vaccine among high-risk urban populations in Southern Thailand using the COM-B Model," provided a clear and accurate presentation, utilizing a web-based cross-sectional approach in the Hat Yai district, Southern Thailand. It strengthened its findings by drawing on a diverse array of contemporary literature, encompassing socio-economic implications, global vaccine acceptance rates, and factors influencing vaccine acceptance. Through an extensive citation of current literature, the research established a robust contextual foundation, weaving insights from reviews on socio-economic impacts, paradigms of vaccine development, and studies on vaccine acceptance rates into its analytical framework. The comprehensive and up-to-date engagement with relevant sources enhanced the study's credibility, providing a well-supported backdrop for its research findings. "I am honored to have the opportunity to read this paper."

**Response:** Thank you so much for your suggestions and comments. Your suggestion and comments have significantly improved our manuscript.

At last, we would like to thank respected editor and respected reviewers for the overall positive feedback and help on the improvement of our manuscript and hope that it will be acceptable to them.

*Sincerely,*
